# Doxorubicin-Loaded Iron Oxide Nanoparticles Induce Oxidative Stress and Cell Cycle Arrest in Breast Cancer Cells

**DOI:** 10.3390/antiox12020237

**Published:** 2023-01-20

**Authors:** Elisa Parcero Hernandes, Danielle Lazarin-Bidóia, Raquel Dosciatti Bini, Celso Vataru Nakamura, Luiz Fernando Cótica, Sueli de Oliveira Silva Lautenschlager

**Affiliations:** 1 Post-Graduate Program in Pharmaceutical Sciences, Universidade Estadual de Maringá, Maringá 87020-900, Paraná, Brazil; 2 Post-Graduate Program in Physics, Universidade Estadual de Maringá, Maringá 87020-900, Paraná, Brazil

**Keywords:** magnetic nanoparticles, doxorubicin, breast cancer, MCF-7 cells

## Abstract

Cancer is one of the most common diseases nowadays and derives from the uncontrollable growth of a single cell. Magnetic nanoparticles (NpMag) offer various possibilities for use in the biomedical area, including drug delivery mediated by magnetic fields. In the current study, we evaluated the in vitro effects of iron-oxide magnetic nanoparticles conjugated with the antitumor drug doxorubicin (Dox) on human breast cancer cells. Our results revealed that magnetic nanoparticles with Dox (NpMag+Dox) induce cellular redox imbalance in MCF-7 cells. We also demonstrate that iron-oxide nanoparticles functionalized with Dox induce oxidative stress evidenced by DNA damage, lipid peroxidation, cell membrane disruption, and loss of mitochondria potential. As a result, NpMag+Dox drives MCF-7 cells to stop the cell cycle and decrease cell migration. The association of NpMg+Dox induced a better delivery of Dox to MCF cells, mainly in the presence of a magnetic field, increasing the death of MCF cells which might reduce the toxicity for healthy cells providing a better efficacy for the treatment. Thus, iron-oxide nanoparticles and doxorubicin conjugated may be candidate for anticancer therapy.

## 1. Introduction

Cancer is one of the most common diseases nowadays and derives from the uncontrollable growth of a single cell [1]. There are many types of cancer with few typical or common characteristics, and its treatment is challenging. The effectiveness of conventional chemotherapy is reduced by the non-specific delivery, rapid clearance, drug resistance, low efficiency, and the significant toxicity of existing antitumor agents once administered at higher doses. Therefore, in the last decades, great efforts have been dedicated to understanding the molecular and cellular mechanisms of this disease, as well as to the production of drugs for its treatment [2].

Magnetic nanoparticles (MNP) offer diverse possibilities for use in the biomedical area due to their unique physicochemical characteristics, including their magnetic properties, as their property of superparamagnetism allows them to be magnetized only under the influence of an external magnetic field, and to lose their magnetization once removed. This behavior of superparamagnetic materials results in potential advantages for the application of therapeutics in specific sites under the influence of an external magnetic field; they can be reverted to their non-magnetic states by removing the external magnetic field, allowing them to be excreted [3,4,5]. Compared to gold and silver nanoparticles, iron oxides are considerably more economical and easier to synthesize, some of which have been approved by the Food and Drug Administration (FDA) as an image contrast agent and anti-iron deficiency drug [6,7,8]. Considering this, iron-oxide nanoparticles have been reported as highly biocompatible nanomaterials which do not pose a serious threat to the organism [9]. In addition, several surface modifications can be used to avoid agglomeration, improve stability, and facilitate connection with other functional groups or drugs. Many studies have shown that these strategies have great potential for targeted drug delivery [10,11,12,13].

Doxorubicin (Dox) is one of the most effective antitumor drugs and acts against several types of tumors. Its molecular mechanism consists of DNA intercalation and inhibition of topoisomerase II enzyme activity [14]. However, its clinical use is severely limited by the cardiotoxicity induced by the generation of reactive oxygen species (ROS) [15]. Therefore, the use of nanocarriers becomes an important mechanism, as it can deliver drugs to specific targets and, consequently, reduce the side effects caused by antitumor drugs in healthy cells [16]. Magnetic nanoparticles have an extensive surface area and the ability to cross biological barriers, which favors their use in drug delivery. Furthermore, recent studies have shown the high efficiency of Dox usage associated with magnetic nanoparticles in the death of breast cancer cells [16,17].

According to this information, in the present study, we evaluated the in vitro effect of iron-oxide nanoparticles functionalized with the antitumor drug doxorubicin in human breast adenocarcinoma cells. Our results show that iron oxide nanoparticles functionalized with doxorubicin were effective in inducing oxidative stress that leads MCF-7 cells to cell cycle arrest and decrease migration.

## 2. Materials and Methods

### 2.1. Nanoparticles Synthesis

Iron oxide nanoparticles (NpMag) were provided by the Multifunction Devices Development Group (GDDM) of the Department of Physics at UEM. NpMag (mean diameter 10 nm) were prepared by the co-precipitation synthesis pathway, fully described in our previous work [18].

### 2.2. Doxorubicin Loading

The load of the anticancer doxorubicin (Dox) on the nanoparticles surface was carried out by suspending the NpMag in an aqueous solution of Dox following an adaptation of the protocol used by S. Kayal et al. [19]. For this, 5 mg of nanoparticles were added to 5 mL of an aqueous solution of Dox 0.1 mg/mL, pH 7.4. This mixture was kept under stirring at 25 °C for 24 h. The loaded nanoparticles, NpMag+Dox, were decanted by centrifugation at 12,000× *g* rpm for 30 min and the supernatant was reserved. For the measure of the loading efficiency (LE) of Dox, a measurement of UV/Vis (480 nm) absorbance of the supernatant was performed on a T90 spectrophotometer from PG Instruments Ltd., Leicestershire, United Kingdom. The drug loading efficiency rate was calculated by the Equation (1):

(1) $$LE\left(\%\right)=\frac{ABS_{total}-ABS_{drug}}{ABS_{Total}}\times 100\;\left[\text{RB1}\right]$$

The resulting nanoparticles, NpMag+Dox, were dispersed in water and reserved. A drop of this solution was spread on a microscope slide and visualized under a fluorescence microscope (Olympus BX51 fluorescence microscope, Tokyo, Japan) as a first indication of the Dox presence in the nanoparticles. The images were recorded with an Olympus UC30 camera, Tokyo, Japan.

### 2.3. Nanoparticles Characterization

Infrared spectra were recorded with a Bruker Vetex FTIR spectrophotometer. Powder samples were ground with KBr and compressed into pellets. FTIR spectra in the 4000–400 cm^−^^1^ range were recorded by accumulating 128 scans, with a resolution of 4 cm^−^^1^. Size distribution and zeta potential (*ζ*) measurements were performed to investigate the size and surface charge of magnetic nanoparticles in aqueous media. The processed nanoparticles were evaluated by a Litesizer 500 analyzer—Anton Paar, in which the *ζ* values were calculated using the Kalliope software version 2.10.6. The measurements were performed by dispersing diluted samples of sonicated magnetite nanoparticle, at room temperature, in deionized water at a concentration of 0.01 mg/mL.

### 2.4. MCF-7 Cells Treatment

Breast adenocarcinoma cells lineage, MCF-7 (ATCC HTB-22; BCRJ—Banco de Células do Rio de Janeiro) were seeded in 6, 12, or 24-well plates at a density of 2.5 × 10^5^ cells/mL, in Dulbecco’s Modified Eagle’s medium (DMEM, Gibco) complete culture medium supplemented with 10% fetal bovine serum (FBS), kept in 37 °C with 5% CO_2_ for 24 h. After washing with phosphate buffered saline (PBS), it was treated with 0.4 and 0.8 µg/mL of free Dox and NpMag and NpMag+Dox at concentrations of 5 and 10 µg/mL and incubated in DMEM complete culture medium, at 37 °C and 5% CO_2_ for 48 h.

### 2.5. Determination the Generation of Total Reactive Oxygen Species (ROS)

Total reactive oxygen species were detected using the 2′ fluorescent probe, 7′-dichlorodihydrofluorescein diacetate (H_2_DCF-DA, Molecular Probes, Leiden, The Netherlands). Intracellular esterases cleave H_2_DCF-DA to H_2_DCF which are oxidized by intracellular ROS to a highly fluorescent DCF product [20]. Thus, after MCF-7 cells treatment with the nanoparticles, the cells were washed, resuspended in PBS and labeled with 5 µM H_2_DCF-DA at 37 °C for 30 min in the dark and then washed once with PBS. The fluorescence was observed on an Olympus BX51 fluorescence microscope (Olympus, Tokyo, Japan) and images were recorded with an Olympus UC30 camera (Olympus, Tokyo, Japan). Moreover, fluorescence was measured in a spectrofluorometer (VICTOR^™^ X3; PerkinElmer, Massachusetts, USA) at 485 nm excitation and 535 nm emission.

### 2.6. Detection of Nitric Oxide

Nitric oxide (NO) was detected using DAF-FM, a non-fluorescent cell permeable probe that reacts with NO to form a fluorescent benzotriazole [21]. Therefore, after MCF-7 cells treatment with the nanoparticles, the cells were loaded with 1 µM DAF-FM diacetate in the dark for 30 min. Then, the cells were washed and resuspended in phosphate buffered saline (PBS). Data acquisition and analysis were performed using a FACSCalibur flow cytometer (Becton-Dickinson, Rutherford, NJ, USA) equipped with CellQuest software (Joseph Trotter, Scripps Research Institute, La Jolla, CA, USA). A total of 10,000 events were acquired.

### 2.7. Reduced Thiols Assay

The thiols content was determined with the 5,5′-dithiobis-(2-nitrobenzoic acid) reagent (Molecular Probes^®^, Eugene, OR, USA), a marker that, in contact with reduced thiols, forms a yellow chromogen that can be detected in a spectrophotometer [22]. Hence, after MCF-7 cells treatment with the nanoparticles, the cells were centrifuged, added in 10 mM Tris-HCl buffer (pH 2.5) and sonicated. Then, the cells were centrifuged and the supernatant was incubated with potassium phosphate buffer (pH 7.5) and 5,5′-dithiobis-(2-nitrobenzoic acid) (1 mM). The reading was performed in a microplate reader (Bio-Tek^®^, Power Wave XS, Winooski, VT, USA) at 412 nm.

### 2.8. DNA Fragmentation

TUNEL Assay Kit detects DNA fragmentation of apoptotic cells by marking DNA breaks using standard immunohistochemical techniques [23]. Thus, after treatment with the nanoparticles, the cells were fixed by incubation in 4% formaldehyde for 30 min. Cells were permeabilized using 0.2% Triton X-100 (in PBS) for 45 min at 37 °C to facilitate staining. All wells received a DNA marking solution (10 µL of reaction buffer, 0.75 µL of TdT enzyme, 8.0 µL of BrdUTP and 31.25 µL of dH_2_O) and incubated for 60 min at 37 °C. Afterwards, 1 mL of rinse buffer was added and the cells were centrifuged. The supernatant was removed and Alexafluor™488 was added. The nuclei were counterstained with propidium iodide (PI). Cells were stored in PBS at 4 °C until analysis and dUTP-labeled DNA was visualized by flow cytometry. Data acquisition and analysis were performed using a FACSCalibur flow cytometer (Becton-Dickinson, Rutherford, NJ, USA) equipped with CellQuest software (Joseph Trotter, Scripps Research Institute, La Jolla, CA, USA). A total of 10,000 events were acquired.

### 2.9. Determination of Lipid Peroxidation

Lipid peroxidation was detected using diphenyl-1-pyrenylphosphine (DPPP) that reacts stoichiometrically with hydroperoxide in lipids, to give diphenyl-1-pyrenylphosphine oxide (DPPP-O), a fluorescent probe [24]. Consequently, after treatment with the nanoparticles, the cells were labeled with DPPP (20 μM) for 30 min at room temperature. Fluorescence intensity was quantified using a fluorescence microplate reader (VICTOR^™^ X3, PerkinElmer) with 380 and 460 nm excitation and emission wavelengths, respectively. Fluorescence intensity was normalized by cell number.

### 2.10. Assessment of Cell Membrane Integrity

Membrane integrity was detected using PI, an impermeable dye that can only cross compromised cell membranes, intercalating with DNA in the nucleus [25]. Hence, after treatment with the nanoparticles, the cells were harvested, centrifuged and resuspended in PBS and incubated with 4 µg/mL of PI for 10 min to verify the cell membrane integrity. Data acquisition and analysis were performed using a FACSCalibur flow cytometer (Becton-Dickinson, Rutherford, NJ, USA) equipped with CellQuest software (Joseph Trotter, Scripps Research Institute, La Jolla, CA, USA). A total of 10,000 events were acquired.

### 2.11. Assessment of Mitochondrial Membrane Potential (*ΔΨm*)

The ΔΨm was monitored by rhodamine 123 (Rh123), a cationic fluorescent red-orange dye. Mitochondrial energization induces suppression of Rh123 fluorescence and the rate of fluorescence decay is proportional to the mitochondrial membrane potential [26]. Thus, after treatment with the nanoparticles, the cells were washed with PBS buffer and marked with a 5 µg/mL solution of Rh123 for 15 min at 37 °C, to verify the ΔΨm. After the initial incubation period with Rh123, the cells were washed and resuspended in PBS for analysis on a FACSCalibur flow cytometer (Becton-Dickinson, Rutherford, NJ, USA) equipped with CellQuest software (Joseph Trotter, Scripps Research Institute, La Jolla, CA, USA). A total of 10,000 events were acquired.

### 2.12. Cell Cycle Analysis

The cell cycle was determined by staining the DNA with PI, allowing the differentiation of cells in G0/G1, S phase, and G2/M [27]. Therefore, after treatment with the nanoparticles, the cells were centrifuged at 1500× *g* rpm for 5 min, collected and resuspended in 70% methanol for fixation. Then, 5 μL of Rnase was added and stained with 5 μL of PI for 30 min at 37 °C. Cell DNA content was analyzed by flow cytometer (FACSCalibur, BD Biosciences). A total of 10,000 events were acquired. Cell cycle data were analyzed and processed on a ModFit LT™.

### 2.13. Wound Healing Assay

The wound healing assay studies cell migration in vitro. This method is based on observing cell migration in a “wound” that is created in a cell monolayer. Then, the rate of wound closure and cell migration can be quantified by photography with an inverted light microscope [28]. Thus, the cells were incubated with 0.5% FBS for 6 h, scratched with a sterile 200 μL pipette tip, washed with PBS and treated with nanoparticles. Cell migration was observed under an inverted phase-contrast microscope (Olympus CKX41; 50× magnification). Data were analyzed using the ImageJ software and calculations for the wound closure percentage (*WC*%) were performed using the following equation:

WC(%)=[(A(0)−A(t))/A(0)]×100

where *A*(0) is the area at time zero (0) and *A*(*t*) is the area after incubation time (*t*). 

### 2.14. Clonogenic Assay

The clonogenic assay is widely used to test the survival of reproductive cell in vitro [29]. The cells ability to form colonies was determined by treating the cells with nanoparticles, after removing the treatments, the cells were washed with PBS, trypsinized and plated at low density (500 cells/well in six-well plates). The cells were cultivated for 15 days and the medium was refreshed every 2–3 days. The colonies were stained with crystal violet. A cell colony was defined as a group formation of at least 50 cells. The plating efficiency (PE) represents the percentage of seeded cells that grow in colonies under a specific culture condition of a given cell line. The survival fraction was calculated as follows: Survival fraction = colonies count/(seeded cells × PE/100).

### 2.15. Evaluation of Magnetic-Field-Mediated Targeted Drug Delivery

To evaluate the magnetic-field-mediated targeted therapy, MCF-7 cells (2.5 × 10^5^ cells/mL) were seeded in T25 flasks with a neodymium magnet attached to the bottom. After 24 h of incubation, cells were treated with nanoparticles in T25 cell culture flasks with a magnet for 48 h. Cells were washed with PBS twice after the incubation time and stained with crystal violet in methanol. Cells were photographed under an inverted phase-contrast microscope (Olympus CKX41; 50× magnification).

### 2.16. Statistical Analysis

The graph shows the average values and standard deviation of three independent experiments. For all tests, statistical analysis was performed by ANOVA (one-way, two-way ANOVA), followed by Tukey test (α = 0.05, *p* < 0.05, *n* = 3). Values of *p* < 0.05 were considered statistically significant. All statistical analysis were performed using Prism 5 software (GraphPad, San Diego, CA, USA).

## 3. Results

### 3.1. Nanoparticle Characterization

In our previous work, the main structural and morphological characterizations of NpMag nanoparticles were carried out and explained in detail [18]. Briefly, the nanoparticles showed an X-ray diffraction (XRD) pattern in accordance with the standard pattern for magnetite. The crystallite size was estimated by the Scherrer’s equation with a value of 10.3 ± 0.8 nm. The transmission electron microscopy (TEM) images show nanoparticles with a shape close to spherical and average diameter of 9.4 ± 0.2 nm obtained by a Log-normal distribution. The value found for the saturation magnetization was 63.8 emu/g obtained by a magnetic hysteresis curve. At room temperature, the sample showed a curve with coercivity and remanence nearly to zero, indicating a superparamagnetic behavior.

The LE obtained for the NpMag+Dox sample was 85% for a concentration of 1 mg/mL of nanoparticles. The effective Dox concentration of 5 and 10µg/mL nanoparticles formulations are 0.392 and 0.783 µg, respectively. This was calculated based on the loading efficiency, using the percentage as the basis for calculation and a Dox calibration curve [18].

**Figure 1:**
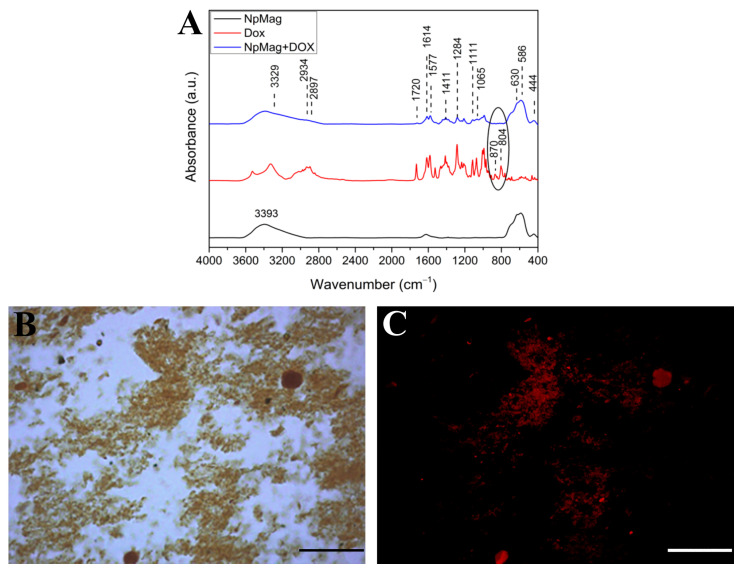
(**A**): FTIR spectrum for NpMag, Dox and NpMag+Dox (**B**): Light and (**C**): fluorescence image of NpMag+Dox. Scale bar: 50 µm.

The FTIR spectra for prepared samples and for pure Dox are presented in Figure 1A. The spectrum for the NpMag+Dox sample shows the characteristic bands with maximum absorbance at 444, 586, and 630 cm^−1^ corresponding to the stretching vibrations of the Fe-O bonds in the magnetite tetrahedral and octahedral crystallographic sites, respectively. Furthermore, characteristic bands of pure Dox appear at 1614, 1577, and 1411 cm^−1^ corresponding to the C=C ring stretch, the bands at 1111, 1065 cm^−1^ correspond to the C–O–C stretch and also at 1720 cm^−1^ band corresponding to the carbonyl group of the Dox molecule [30,31]. The bands observed at 805 and 870 cm^−1^ in the pure Dox spectrum refer to the N-H waggin vibration. In the conjugation spectrum, these bands decrease, suggesting that the interaction of Dox with the surface of nanoparticles occurs via -NH_2_ groups with -OH groups on the magnetite surface through electrostatic interactions [19,32,33].

Figure 1B,C show the light and fluorescence microscopy images, respectively, for the NpMag+Dox sample. The images show fluorescence in the red region due to Dox excitation, indicating its presence in nanoparticles. The mean hydrodynamic radius (R_H_), the polydispersity index (PDI), and the zeta potential (ζ) for the samples are shown in Table 1. The increase in the hydrodynamic radius in the NpMag+Dox sample indicates the presence of the Dox molecule on the surface of the nanoparticles when compared to the pure sample. The PDI values, both >10%, indicate that the samples are moderately polydispersed. The ζ values indicate a low stability of samples dispersed in water [34]. However, an increase in surface charge from −9.6 to −0.7 mV strongly suggested that positively charged Dox molecules are bound to negatively charged nanoparticles through electrostatic interactions [35].

### 3.2. In Vitro Biological Assays

Our aim in the next set of results is to show how NpMag+Dox induces oxidative stress in MCF-7 cells compared to control and compared to Dox alone. The toxicity of NpMag-Dox has already been tested, showing that this association did not induce toxicity in HaCat cells at the tested concentration [18].

#### 3.2.1. NpMag+Dox Increases the Reactive Oxygen and Nitrogen Species in MCF-7 Cells

**Figure 2:**
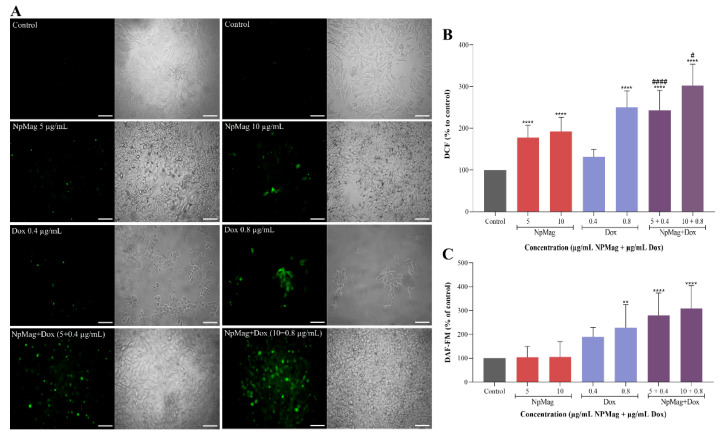
ROS generation measurement by fluorescence microscopy (**A**) and spectrofluorometer (**B**) using H2DCF-DA; detection of nitric oxide using DAF-FM (**C**) in MCF-7 cells treated with 5 and 10 µg/mL of NpMag and 0.4, and 0.8 µg/mL of Dox and NpMag+Dox for 48 h. Scale bar: 1 µm. ** *p* < 0.01, **** *p* < 0.0001 significant difference compared to the control. # *p* < 0.05, #### *p* < 0.0001 significant difference compared to the same concentration of Dox.

According to Figure 2A, control cells showed a low fluorescence intensity, indicating absence of ROS production. In the group of MCF-7 cells treated with NpMag conjugated with Dox, the fluorescence intensity increased in comparison to the control, Dox alone, and NpMag alone, indicating ROS production. In cells treated only with Dox or only with NpMag, at both concentrations tested, the fluorescence intensity also increased, compared to the control. Similar results were observed in Figure 2B. The treatment of MCF-7 cells with nanoparticles functionalized with Dox (0.4 and 0.8 µg/mL) increased ROS formation at 48 h (107% and 151%, respectively), (Figure 2B), compared to the control. NpMag+Dox 0.4 and 0.8 mg/mL also induced an increase in ROS (82% and 13%, respectively), compared to the Dox alone at the same concentrations. Dox 0.8 µg/mL increased ROS production (122%), compared to the control. Synthesized NpMag at 5 and 10 µg/mL showed a significant increase (55% and 64%, respectively) in ROS, compared to the control.

Figure 2C shows that the treatment of MCF-7 cells with 0.4 and 0.8 µg/mL of NpMag+Dox significantly increased NO levels (168% and 218%, respectively), compared to the control. Dox at concentrations of 0.8 µg/mL induced an increase (132%) in NO levels, compared to the control. For NpMag concentrations, 5 and 10 µg/mL did not induce increase in reactive nitrogen species (RNS).

#### 3.2.2. NpMag+Dox Decreases the Levels of Reduced Thiols in MCF-7 Cells

**Figure 3:**
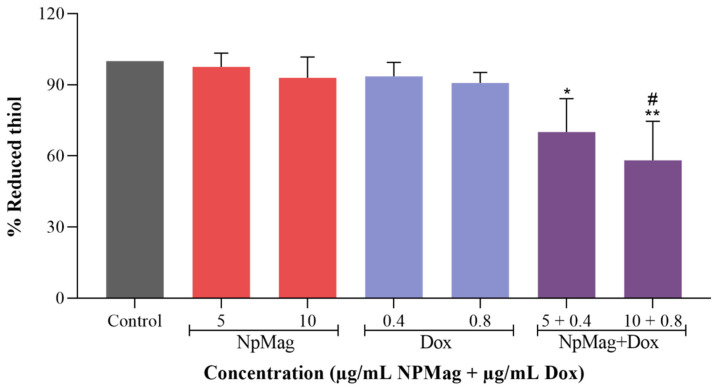
Production of reduced thiols levels by the DTNB assay in MCF-7 cells treated with 5 and 10 µg/mL of NpMag and 0.4 and 0.8 µg/mL of Dox and NpMag+Dox for 48 h. * *p* < 0.05, ** *p* < 0.01 significant difference compared to the control. # *p* < 0.05 significant difference compared with to same concentration of Dox.

As shown in Figure 3, NpMag+Dox induced a significant decrease (30% and 42%) in reduced thiols levels at concentrations of 0.4 and 0.8 µg/mL in 48 h, compared to the control. NpMag+Dox 0.8 µg/mL also induced a significant decrease in reduced thiols levels (36%), compared to the Dox at the same concentration. Dox and NpMag did not induce any reduction in the reduced thiols levels, compared to the control.

#### 3.2.3. NpMag+Dox Induces DNA Damage, Increases Lipid Peroxidation, Induces Membrane Integrity Loss, and Decreases ΔΨm in MCF-7 Cells

**Figure 4:**
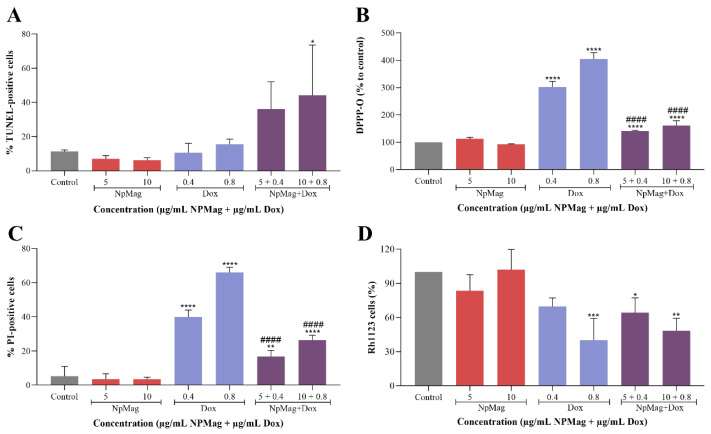
DNA fragmentation using the TUNEL assay (**A**); production of lipid peroxidation by spectrofluorometer using DPPP (**B**); membrane integrity using PI fluorescence (**C**); and mitochondrial membrane potential using Rh123 (**D**) in MCF-7 cells treated with 5 and 10 µg/mL of NpMag, NpMag+Dox, and 0.4 and 0.8 µg/mL of Dox and NpMag+Dox for 48 h. * *p* < 0.05, ** *p* < 0.01, *** *p* < 0.001, **** *p* < 0.0001 significant difference compared to the control. #### *p* < 0.0001 significant difference compared with to same concentration of Dox.

Figure 4A shows that NpMag+Dox 0.8 µg/mL induced a significant increase (289%) in DNA fragmentation compared to the control. The NpMag and Dox did not induce any DNA fragmentation compared to the control.

Figure 4B shows that NpMag+Dox, at both concentrations tested, induced a significant increase (41% and 60%, respectively) in lipid peroxidation compared to the control. However, NpMag+Dox 0.4 and 0.8 µg/mL induced a significant decrease (53% and 60%, respectively) in lipid peroxidation compared to the same concentration of Dox. Concentrations of 0.4 and 0.8 µg/mL of Dox induced a significant increase (202% and 303%, respectively) in lipid peroxidation compared to the control. NpMag alone did not induce any alteration in lipid peroxidation compared to the control.

In Figure 4C we can observe that NpMag+Dox, at both tested concentrations, induced a significant increase (higher than 215%) in membrane disruption compared to the control. However, NpMag+Dox 0.4 and 0.8 µg/mL induced a significant decrease (58 and 60% respectively) in membrane disruption compared to the same concentration of Dox. The effect of Dox treatment was dose-dependent with a significant increase (higher than 650%) in membrane disruption compared to the control. NpMag alone did not induce any membrane disruption.

Flow cytometry demonstrated in Figure 4D shows that the NpMag+Dox sample decreased (36% and 52%, respectively) the ΔΨm at the concentration of 0.4 and 0.8 µg/mL compared only to the control. Dox at concentrations 0.8 µg/mL significantly decreased (60%) the ΔΨm compared to control. NpMag did not induce any alteration in ΔΨm.

#### 3.2.4. NpMag+Dox Increases G2 Cell Cycle Arrest in MCF7 Cells

**Figure 5:**
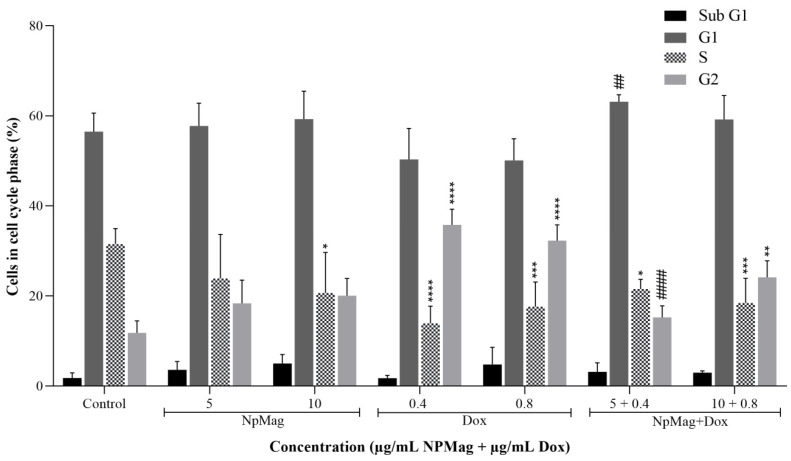
Cell cycle phases by PI fluorescence in breast cancer cells, MCF-7, treated with 5 and 10 µg/mL of NpMag and 0.4 and 0.8 µg/mL of Dox and NpMag+Dox. * *p* < 0.05, ** *p* < 0.01, *** *p* < 0.001, **** *p* < 0.0001 compared to the control. ## *p* < 0.01, #### *p* < 0.0001 significant difference compared to the same concentration of Dox.

As shown in Figure 5, NpMag+Dox at 0.8 µg/mL induced a significant increase in the cells population in the G2 phase (104%), compared to the control. NpMag+Dox 0.4 µg/mL induced a significant decrease in the G2 phase (57%), compared to the same concentration of Dox. All tested concentrations of Dox induced an increase in the G2 phase (higher than 170%), compared to the control. NpMag did not induce any alteration in the G2 phase, compared to the control. In the S-phase, a significant decrease in cell population was observed after treatment with NpMag+Dox 0.4 and 0.8 µg/mL (32% and 41%, respectively), compared to the control. Dox 0.4 and 0.8 µg/mL also induced a significant decrease (higher than 40%) in the S-phase cell population, compared to the control. NpMag-treated cells at 10 µg/mL exhibited a significant decrease in the S phase (34%), compared to the control.

#### 3.2.5. NpMag+Dox Inhibits MCF7 Cells Migration

**Figure 6:**
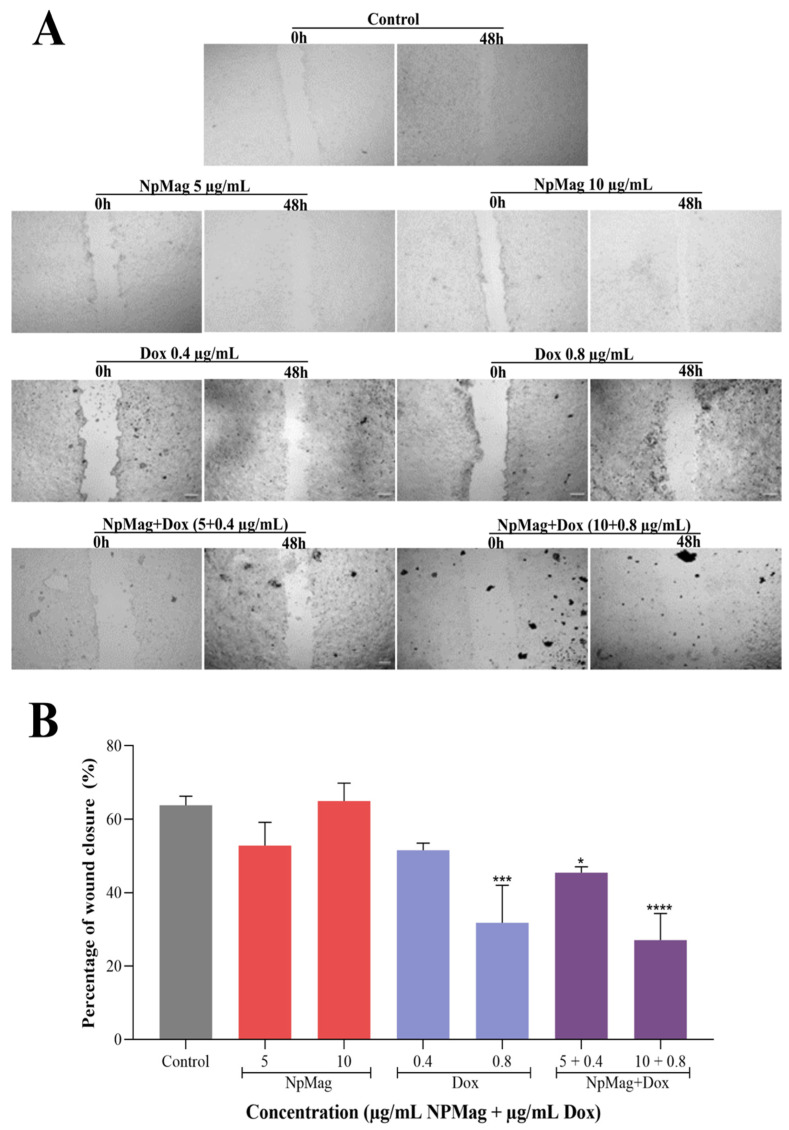
(**A**): Analysis of cell migration from the wound healing assay images. (**B**): Percentages of in vitro closure area by the wound healing assay (**B**) after 48 h of MCF-7 cells treated with 5 and 10 µg/mL of NpMag and 0.4 and 0.8 µg/mL of Dox and NpMag+Dox. Scale bar: 200 µm. * *p* < 0.001, *** *p* < 0.001, **** *p* < 0.0001 significant difference compared to the control.

The wound healing assay demonstrated in Figure 6A,B that control cells closed 64% scratched wounds within 48 h. NpMag+Dox 0.4 and 0.8 µg/mL inhibited wound closure for 48 h (29% and 58%, respectively), compared to the control (Figure 6A,B). Dox inhibited wound closure at concentrations of 0.8 µg/mL (50%), compared to the control. NpMag did not inhibit cell migration, compared to the control.

#### 3.2.6. NpMag+Dox Inhibits MCF7 Cells Colonies Formation

**Figure 7:**
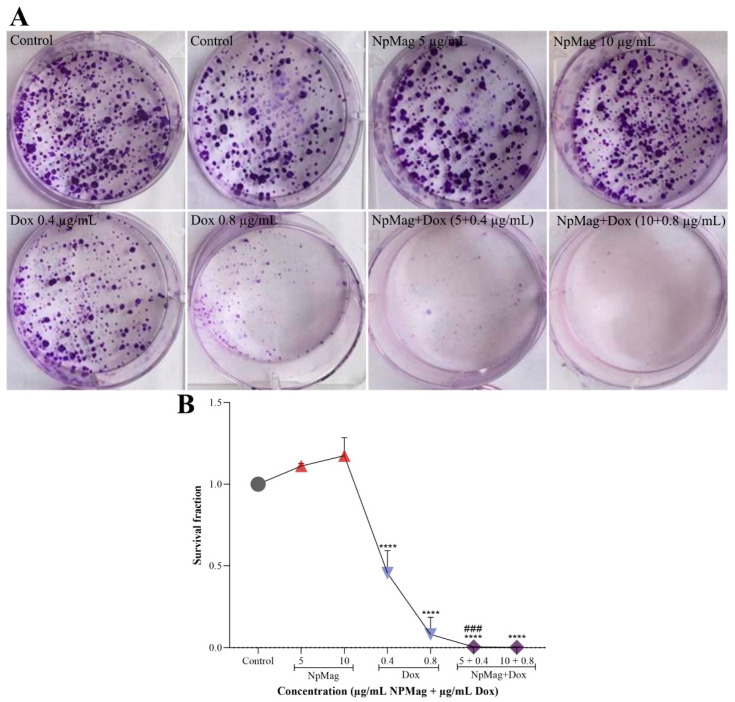
Surviving fraction of the clonogenic assay in breast cancer cells, MCF-7, treated with 5 and 10 µg/mL of NpMag and 0.4 and 0.8 µg/mL of Dox and NpMag+Dox. (**A**): Representative images and, (**B**): Survival fraction graph. **** *p* < 0.0001 significant difference compared to the control. ### *p* < 0.001 significant difference compared to the same concentration of Dox.

In Figure 7 we can observe that NpMag+Dox, at both tested concentrations, showed a significant decrease in the capacity of cells to generate new colonies (99% and 100%, respectively), compared to the control. NpMag+Dox 0.4 µg/mL also induced a significant decrease (99%) in the capacity of cells to generate new colonies, compared to Dox at the same concentration. Dox alone at 0.4 and 0.8 µg/mL induced a significant decrease in colony formation (54% and 92%, respectively), compared to the control. NpMag alone did not induce a decrease in cell survival, compared to the control.

#### 3.2.7. NpMag+Dox Inhibits MCF-7 Cells Grow in the Region of Magnetic Field

**Figure 8:**
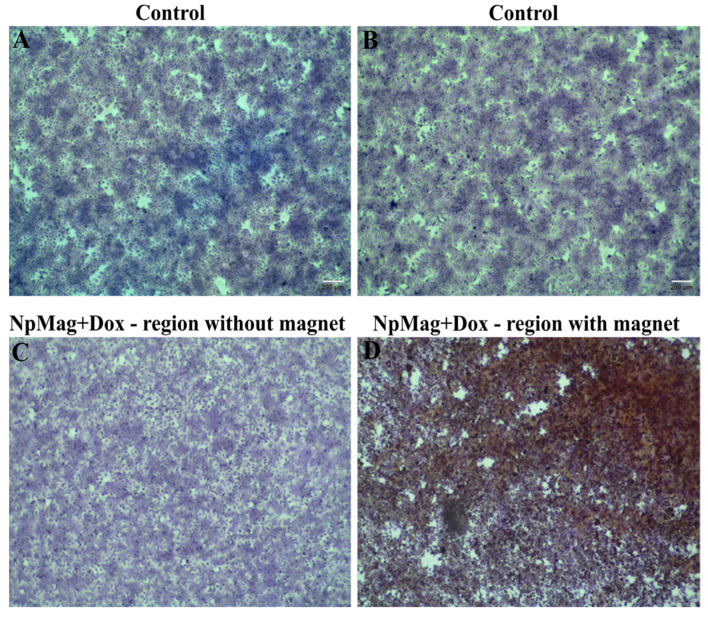
Magnetic-field-mediated drug delivery: (**A**,**B**) Bright-field images of crystal violet stained MCF-7 cells control without treatment; (**C**) MCF-7 cells treated with NpMag+Dox region without magnet and (**D**) MCF-7 cells treated with NpMag+Dox region with magnet. Scale bar: 200 µm.

Figure 8 shows the treatment efficiency of MCF-7 cells with NpMag+Dox through microscopic analyses that clearly revealed morphological changes and a detachment of MCF-7 cells after treatment with NpMag+Dox in the region where the magnet was placed, as shown in Figure 8D, compared to the control.

## 4. Discussion

Conventional treatment for most cancer cells lacks efficacy and induces significant toxicity. There are innumerous efforts to develop new cancer treatments with better efficacy, safety, and selectivity [36]. Here we show the in vitro effect of iron-oxide nanoparticles functionalized with the antitumor drug doxorubicin on the cellular redox state of human breast adenocarcinoma cells and we also draw a possible mechanism that leads breast cancer cells to interrupt the cell cycle and decrease the migration.

We initially reported the preparation and characterization of NpMag [18]. These magnetic nanoparticles are composed of iron-oxide, which is biodegradable, biocompatible, and demonstrates superior chemical stability compared to other metallic nanoparticles [37].

We then showed that magnetic nanoparticles induced oxidative stress in MCF-7 cells, evidenced here by an even greater increase in ROS than treatment with Dox alone. Reactive oxygen species are molecules that cause oxidative damage/stress in cellular models [38]. We also showed that magnetic nanoparticles with Dox induced a significant increase in NO levels, which is also a free radical with a very complex biological function. This radical may be a biological messenger used by cells to prevent intracellular damages caused by ROS. In addition, NO has a high affinity for metals and many biological effects can be attributed to its chemical interaction with iron: for example, activation of guanylyl cyclase appears to be mediated by nitrosylation of heme iron [39] and Fe–S clusters are decomposed after interaction with NO [40]. The state of oxidative stress in cells plays an important role in cellular signaling and in inflammatory, genotoxic, and proliferative responses [41]. In fact, it has been reported that iron-oxide nanoparticles generate redox imbalance in cancer cells, mainly by producing ROS [42]. In addition, Dox can directly interact with iron to form reactive anthracycline-iron complexes, resulting in an iron cycle between Fe^3+^ and Fe^2+^ associated with ROS production, including the high-toxic hydroxyl radical (OH•) by Fenton and Haber–Weiss reactions, consequently altering homeostasis, leading to increased cell death [43].

As expected, NpMag+Dox decreased reduced thiol levels in MCF-7 cells that are present in endogenous antioxidant enzymes that can prevent damage to cellular components caused by oxidative stress. This reduction may involve the intracellular antioxidant enzyme impairment, mainly related to the high increase in total ROS and RNS induced by nanoparticles functionalized with Dox. However, reduced thiol levels did not decrease for the same concentrations of Dox alone, probably due to an increase in the presence of reduced thiols by the tumor cell, a sign of resistance in tumor cells [44].

As a result of the overall increase in ROS and RNS formation and the decrease in antioxidant enzyme activity, the treatment of MCF-7 cells with magnetic nanoparticles with Dox alters the redox balance inducing cellular oxidative stress. Consequently, we observe DNA damage, lipid peroxidation, and loss of membrane integrity. Interestingly, DNA damage was more pronounced with magnetic nanoparticles with Dox than Dox alone, suggesting DNA may be a target of nanoparticles association with Dox leading to cell cycle arrest. As is known, the extensive fragmentation of genomic DNA generates a multitude of DNA double-strand breaks (DSBs). Indeed, it has been demonstrated that the effect of magnetic nanoparticles targets the DNA involved in the cell cycle of cancer cells [45]. The rate of nanoparticle entrance into cells is slower than Dox, which may explain why some consequences of oxidative stress, such as lipid peroxidation and membrane integrity, are less prominent in cells treated with NpMag+Dox. The association of NpMag+Dox also induced loss of mitochondria potential. Loss of ΔΨm is a sign of bioenergetics stress and may result in disruption of essential cells biochemical and molecular process.

As a consequence, we can say that nanoparticles with Dox induced imbalance in the cellular redox system above the cellular tolerability threshold, leading to cell cycle arrest and inhibiting MCF-7 cell migration and colony formation. The G2 checkpoint prevents cells from entering mitosis when DNA is damaged, providing an opportunity to repair and stop the proliferation of damaged cells [46]. In the literature, we found the influence of iron-oxide nanoparticles on the cell cycle of cancer cells [45]. Moreover, depending on the size of nanoparticles, S-phase arrest can be induced [47]. Here, as expected, we observed an increase in the G1 and S-phase, showing that nanoparticles with Dox do not interfere only in the G2 phase [48]. It is important to emphasize that free Dox passes from the extracellular matrix to the intracellular matrix by simple passive diffusion and easily reaches the nucleus. In the case of NpMag with Dox, the drug is released from the iron-oxide nanoparticle surface in the lysosomes due to the acidic pH environment and then the Dox can reach the nucleus and intercalate the DNA, which may take longer than the Dox alone to obtain the same or better effect [49]. In cancer cells, the ability of cells to migrate is essential for many processes, including tumor invasion, neoangiogenesis, and metastasis [50]. Finally, we showed that these magnetic nanoparticles with Dox were able to induce MCF-7 cells to die in the region of a magnet field. In this assay, the presence of a magnetic field guides and retains the magnetic nanoparticles, enabling the release of Dox locally. Other studies, as shown in Unnikrishnan, et al., 2021 [51], used similar approaches to confirm this targeting ability of magnetic nanoparticles, showing that it can be used as a magnetic-field-mediated therapy.

All these together show that Dox-loaded magnetic nanoparticles might be better internalized by MCF-7 cancer cells, leading to a controlled release of a high concentration of Dox inside the cancer cell, inducing toxicity and decreasing MCF-7 cancer cell viability. We know that nanoparticles coated with different materials seem to improve the internalization and the effect of Dox in MCF-7 cells [18,43,52,53,54]. Therefore, alterations in formulations should be considered to enhance biocompatibility and Dox adsorption on the surface of nanoparticles and induce better cytotoxic effects.

## 5. Conclusions

**Figure 9:**
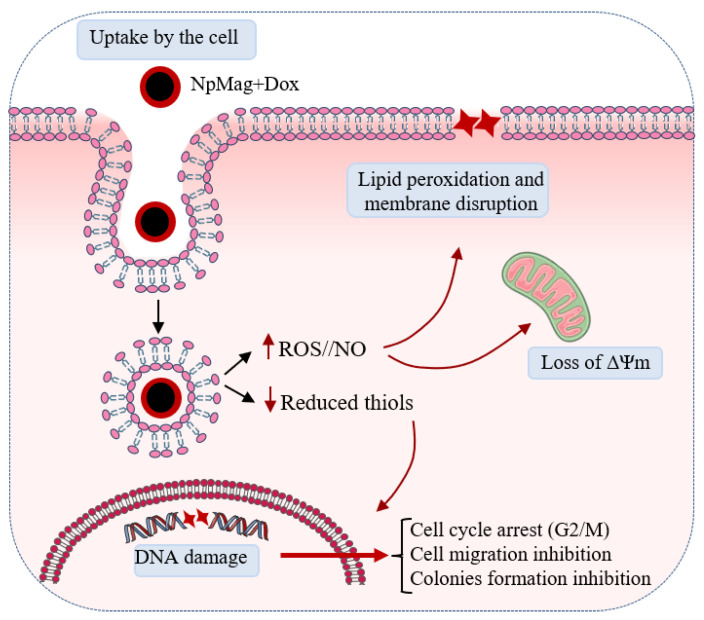
Nanoparticles conjugated with Dox induce oxidative stress and cell cycle arrest, followed by decreased cell migration and colony formation.

Our results showed that Dox-loaded magnetic nanoparticles induce oxidative stress in MCF-7 cells evidenced here by a set of results that, together, lead breast cancer cells to interrupt the cell cycle and decrease migration (Figure 9). This association seems to improve Dox delivery, mainly in the presence of a magnetic field, which might reduce toxicity and provide better efficacy for the treatment. In fact, iron-oxide nanoparticles and doxorubicin association is a candidate for an antitumor drug that has great potential as a promising agent for anticancer therapy.

## Figures and Tables

**Table 1:** The physicochemical characteristics of Fe_3_O_4_ nanoparticles.

Nanoparticle	Hydrodynamic Radius by DLS (nm)	Polydispersity Index (PDI)	Zeta Potential (mV)
NpMag (Fe_3_O_4_)	125.7 ± 30.9	0.17 ± 0.10	−9.6 ± 0.5
NpMag+Dox	193.2 ± 34	0.20 ± 0.03	−0.7 ± 0.1

## Data Availability

All data are contained in the article.
